# Mental health status of early married girls during the COVID-19 pandemic: A study in the southwestern region of Bangladesh

**DOI:** 10.3389/fpsyt.2022.1074208

**Published:** 2023-01-05

**Authors:** Jannatul Ferdous Nishat, Taufiq-E-Ahmed Shovo, Benojir Ahammed, Md. Akhtarul Islam, Mohammad Mizanur Rahman, Md. Tanvir Hossain

**Affiliations:** ^1^Sociology Discipline, Social Science School, Khulna University, Khulna, Bangladesh; ^2^Statistics Discipline, Science, Engineering and Technology School, Khulna University, Khulna, Bangladesh; ^3^Department of Urban and Regional Planning, Jahangirnagar University, Savar, Bangladesh

**Keywords:** mental health, depression, anxiety, stress, DASS-21, early marriage, COVID-19, Bangladesh

## Abstract

**Background:**

Due to unemployment, the prolonged lockdown during the COVID-19 pandemic caused panic and deepened poverty, especially among lower-class and marginal people. The related financial crises led to harmful practices such as the early marriage of adolescent girls, which deteriorated these girl’s mental state.

**Aims:**

This study attempted to assess the prevalence of mental health problems among early married girls and determine the associated predictors of the growing mental health burden.

**Methods:**

This cross-sectional survey was conducted during the third wave of the COVID-19 pandemic in Dumuria Upazila in the Khulna district of Bangladesh. Data were collected purposively from 304 girls who were married off during the COVID-19 pandemic, this was carried out between 22 July and 31 August 2022 by administering a semi-structured interview schedule, with mental health measured by the depression, anxiety, and stress scale 21 (DASS 21). The data were analyzed using IBM SPSS Statistics (version 25), and multiple linear regression was executed in order to predict mental health problems among early married girls.

**Results:**

The findings show that the overall prevalence of depression, anxiety, and stress among early married girls during the COVID-19 pandemic in Bangladesh was 60.9% (95% CI: 0.554–0.663), 74.7% (95% CI: 0.698–0.796), and 23.7% (95% CI: 0.189–0.285). The prevalence was relatively higher among girls from the *Sanatan* (Hindu) religion and younger girls than among Muslim and older girls, respectively. The multiple linear regressions indicate that age, age at marriage, duration of the marriage, spousal occupation, intimate partner violence (IPV), and subjective happiness were the critical predictors of mental health problems among early married girls.

**Conclusion:**

Early marriage, along with various adverse outcomes, i.e., IPV, maladjustment, and poor subjective happiness, has resulted in heightened mental health problems for young girls. Policymakers should implement coercive measures to prevent early marriage, especially during social, economic, political, and health crises; in addition, more research is recommended in order to explore the mechanisms that make early married girls psychologically vulnerable and thus formulate protective and preventive programs for addressing such vulnerabilities.

## Introduction

The outbreak of coronavirus disease (COVID-19) in late 2019 in China led to havoc across the world, resulting in the loss of over 6.5 million people; around 610 million have been infected globally in the subsequent years (1). In Bangladesh, the number of deaths from COVID-19 was around 30,000, the confirmed cases in the country numbered 0.2 million (2). The absence of therapeutic measures compelled the World Health Organization–the global watchdog of health issues–to declare a global pandemic on 11 March 2020 (3) and to advocate the most stringent non-therapeutic measures, including “lockdown,” “social distancing,” “face mask,” and “work from home” for the general population as well as longer-term “isolation” and “quarantine” for those infected with or suspected of having COVID-19 in order to curb the risk of “human-to-human” infection (4, 5). This prolonged “home confinement” as well as the constant “fear of infection” adversely affected the mental health of people globally (6–8). In Bangladesh, there was a spike in mental health disorders during the COVID-19-induced lockdown. For example, a study in April 2020 indicated that university students were suffering from depression (45.9%), anxiety (51%), sleep disturbance (27.1%), and fear of COVID-19 (86%) during the early stage of the COVID-19 pandemic. Another study in May 2020 found that the prevalence of depression and anxiety was 82.3 and 81.8%, respectively, among university students (9). Teachers were also experiencing heightened mental health problems during the pandemic (10). The existing literature suggests that exposure to “misinformation” through social and electronic media (11), uncertainty over academic and professional careers (12), and financial constraints (13–16) were the key factors for growing mental disorders among the adult population.

In addition to affecting mental health conditions, the pandemic has also increased various harmful practices, particularly in developing countries; early marriage is one of these. A report in late 2020 stated that due to the pandemic, around 1.3 to 2.5 million underage girls were at risk of being married off over the next five years (17). Another report, by the United Nations Population Fund (UNFPA), estimated that an additional 13 million early marriages would occur between 2020 and 2030 due to the COVID-19 pandemic (18). In South Asia, around 0.2 million girls are expected to become child brides (19). Maharashtra in India, for example, witnessed a 78% spike in early marriage (20), while in Bangladesh, more than 1,000 early marriages were reported in different regions between March and June 2020 –the first wave of the COVID-19 pandemic (21). The intensified poverty and financial crises caused by COVID-19 have forced many families to have their underage girls married off (20, 21). A study on fishermen in the southwestern regions of Bangladesh indicated that people were forced to marry off their school-going daughters in order to reduce the burden on their households (15), as the most underprivileged and marginalized people were struggling to afford basic amenities, i.e., expenditure for food, clothing, and education, due to job loss (14, 16, 20–22). The immediate consequence of early marriage is multifaceted. Studies indicate that early married girls are subject to different types of violence, including physical, sexual, and emotional (23). In addition, coercive sex by the partner and unwanted pregnancy at an immature age can also lead to various health complexities and loss of life (24). In fact, some girls suffer from extensive traumatic experiences, such as marital maladjustment and psychological distress (24–26), which may lead them to suicide due to the unhappiness in their lives (27).

Although there are studies that have investigated mental health issues and their predictors among adolescents during the COVID-19 pandemic in Bangladesh (28) and other developed and developing countries (29) such as China (30), the United States (31), and the United Kingdom (32), no study to the best of the authors’ knowledge has attempted to ascertain the mental health of early married girls. This study, therefore, is designed to assess the prevalence and possible predictors of mental health problems among early married girls in Bangladesh during the COVID-19 pandemic. The study will help policymakers to identify the potential predictors of mental health problems among early married girls and to devise remedies, i.e., social, economic, legal, cultural, and psychological, in order to prevent mental health burdens on girls and deter possible early marriage through awareness at family and community levels.

## Materials and methods

### Study settings and participants

This cross-sectional study was carried out in Dumuria Upazila, a sub-district of the Khulna district in Bangladesh. Khulna is the southwestern regional hub; it has a population of 2.32 million, of which 66.5% reside in rural areas (33, 34). Among the 14 Upazila in the Khulna district, Dumuria is the most populated (0.31 million) and geographically the second largest (454.2 km^2^) Upazila (34, 35). During the COVID-19 pandemic, over 3,000 schoolgirls, mostly in Class VII and Class X, became child brides in the Khulna district alone. The highest number of these child marriages (751) took place in Dumuria Upazila (36); therefore, it was selected as the study area (see Supplementary Figure 1). However, some specifications were set out when selecting the participants: each participant must be (i) a girl; (ii) married off under 18 years of age during the COVID-19 pandemic; and (iii) a resident of Dumuria Upazila. Considering these criteria, the data were collected purposively using a semi-structured interview schedule from a total of 330 early married girls, out of which 304 were retained for this study following careful scrutiny of the responses.

### Ethical clearance

This study was approved by the institutional ethical clearance committee (Reference No. KUECC–2022/08/24). The participants responded to this cross-sectional study by filling out a written informed consent form in the first section of the interview schedule, following verbal assent from their guardians, i.e., parents, spouse, or in-laws. All participants responding voluntarily to the interview schedule were provided with information in the consent form concerning the research purpose, anonymity, confidentiality of information, and the right to revoke participation without prior justification.

### Procedures

In this study, the data were collected by administering a semi-structured interview schedule containing nine mutually inclusive modules; these were designed following careful review of the relevant literature. Each module had specific questions: modules one, two, three, and four consisted of socio-demographic information about the participants, their parents, their spouses, and their in-laws, respectively; module five highlighted the possible reasons for early marriage; and modules six, seven, eight, and nine focused on intimate partner violence (IPV), marital adjustment, mental health issues, and subjective happiness, respectively. The data were collected in the home settings of the participants by a group of 12 trained data enumerators, during the period from 22 July to 31 August 2022; each interview lasted for around 25 min.

### Measures

#### Socio-demographic information

In this study, the socio-demographic information included age (in years), religion (“Islam” or “*Sanatan* (Hindu)”), education (years of schooling), age at first marriage (in years), duration of marriage (in years), whether the participants had dropped out of school (“yes” or “no”), age of spouse (in years), education of spouse (years of schooling), occupation of spouse (“manual labor/farmer” or “service/business”), and income of spouse (monthly in BDT).

#### Marital adjustment scale

The martial adjustment of the early married girls was assessed by the short marital adjustment scale (SMAS) developed by Locke and Wallace (37) and adapted for Bangladeshi couples by Khatun, Deeba (38). The SMAS is a 15-item self-reported measure that can be used for both partners or just one partner (37). Item 1 measures marital happiness on a seven-point response scale, items 2 to 8 were measured on a six-point scale, and items 9 to item 15 were measured by a tailored scale for each item (37). The overall internal consistency of SMAS was Cronbach’s α = 0.723.

#### Intimate partner violence

The IPV scale was drawn from the World Health Organization’s multi-country study on women’s health and domestic violence (39). The IPV scale comprised of 12 items using a five-point Likert scale, where “1” = never, “2” = rarely, “3” = sometimes, “4” = very often, and “5” = always. It measured physical (5 items), sexual (3 items) and emotional (4 items) violence, and a higher score reflects frequent violence by an intimate partner. The overall internal consistency of the IPV scale in this study was Cronbach’s α = 0.929.

#### Subjective happiness scale

The subjective happiness of the early married girls was measured by the subjective happiness scale (SHS) developed by Lyubomirsky and Lepper (40). The SHS consisted of four items on a seven-point Likert scale; a higher composite score from the SHS reflects greater happiness. The overall internal consistency of SHS was Cronbach’s α = 0.897, while the internal consistency of the original SHS varied from 0.79 to 0.94 based on culture, language, and occupation (40).

#### Depression, anxiety, and stress scale 21

The mental health of early married girls was measured by the widely used depression, anxiety, and stress scale 21 (DASS 21) developed by Lovibond and Lovibond (41). The initial DASS assessed the symptoms of depression, anxiety, and stress and consisted of 42 items, measured on a four-point Likert scale, with 14 items used for each sub-scale (41). Later, Henry and Crawford (42) developed a shorter version–the DASS 21–with seven items used for each sub-scale. The sum of scores of the seven items for each sub-scale was estimated to indicate the presence of negative emotional states, i.e., depression, anxiety, and stress. A score ≥10 of indicates the presence of depression symptoms, a score of ≥8 reflects the presence of anxiety symptoms, and a score of ≥15 signifies the presence of stress symptoms among the participants. It is important to note that DASS 21 is a suitable and reliable scale for measuring the symptoms of mental health problems among adolescents (43, 44). The overall Cronbach’s α (alpha) of DASS 21 in this study was 0.931, reflecting an excellent internal consistency (45), and the internal consistency of each sub-scale was Cronbach’s α = 0.820, Cronbach’s α = 0.817, and Cronbach’s α = 0.795 for depression, anxiety, and stress, respectively.

### Analysis

The data were analyzed in two consecutive phases using IBM SPSS Statistics (Version 25) for Windows. Descriptive statistics, i.e., frequency and percentage analysis, were calculated to present the socio-demographic information of the participants. The prevalence of depression, anxiety, and stress was estimated at a 95% confidence interval (CI). Simple linear regression (SLR) and multiple linear regression (MLR) analysis with unstandardized (B) and standardized Coefficient (β), at 95% CI, were utilized to identify the risk factors associated with mental health problems among early married girls, i.e., depression, anxiety, and stress. The different factors were statistically significant when the *p*-value was <0.05.

## Results

### Socio-demographic information

Table 1 shows the socio-demographic information of the early married girls. The average age of the participants was 17.1 years (±1.42), and more than 70% of the participants were Muslim (73.7%). With an average length of schooling of around 5 years (±3.41), the participants were married off, on average, at the age of 15 years (±1.24). Regarding the girls’ spouses, it is apparent that the average age of the spouse was 25.8 years (±3.64), with around 9 years of schooling on average (±3.15). More than half of the spouses were engaged in manual labor or farming (55.6%); their monthly average income was BDT 16,591 (±6,175.49).

**TABLE 1:** Socio-demographic information of the participants.

Variables	*f* (%)	Statistics *M* and SD	Max–Min
Age		17.1 and 1.42	19–13
Religion			
*Sanatan* (Hindu)	80 (26.3)		
Islam	224 (73.7)		
Education		5.1 and 3.41	9–0
Age at first marriage		15.3 and 1.24	17–12
Duration of marriage		2.0 and 0.74	3–1
Age of spouse		25.8 and 3.64	36–19
Education of spouse		8.9 and 3.15	17–0
Occupation of spouse			
Manual labor/farmer	169 (55.6)		
Service/business	135 (44.4)		
Monthly income of spouse		16592.1 and 6175.49	40,000–8,000

*f*, frequency; *M*, mean; *SD*, standard deviation; Max, maximum; Min, minimum; Number in the parentheses are percentage.

### Prevalence of depression, anxiety, and stress among early married girls

**FIGURE 1:**
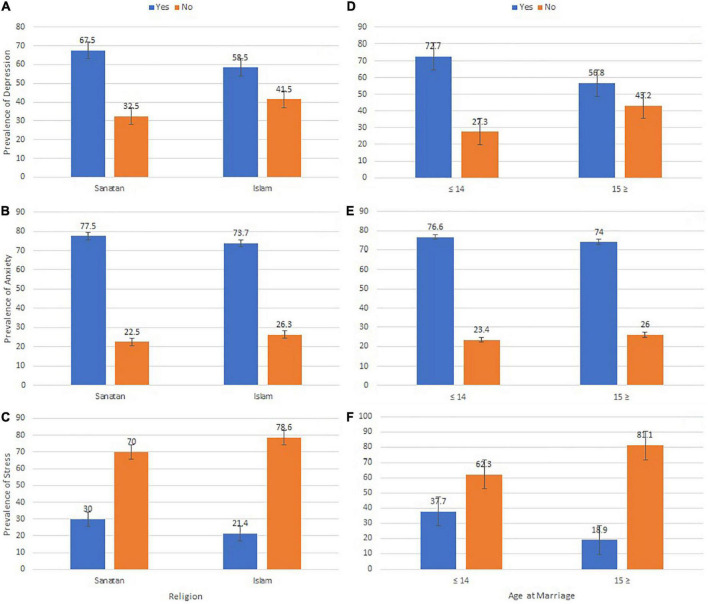
Prevalence and standard error of depression **(A,D)**, anxiety **(B,E)**, and stress **(C,F)** among early married girls in Bangladesh.

The overall prevalence of depression, anxiety, and stress among early married girls in Bangladesh was 60.9% (95% CI: 0.554–0.663), 74.7% (95% CI: 0.698–0.796), and 23.7% (95% CI: 0.189–0.285). The prevalence of depression (72.7 versus 56.8%), anxiety (76.6 versus 74%), and stress (37.7 versus 18.9%) among girls married at or before 14 years of age was higher than in girls married at or after 15 years of age (see Figure 1). Likewise, the prevalence of depression (67.5 versus 58.5%), anxiety (77.5 versus 73.7%), and stress (30 versus 21.4%) was higher among *Sanatan* (Hindu) girls than among Muslim girls.

### Determinants of depression among early married girls

Table 2 shows the SLR and MLR models with unstandardized and standardized coefficients with 95% CI. In the MLR model, the age, religion, age at first marriage, duration of the marriage, income of spouse, marital adjustment, IPV, and subjective happiness of the participants were found to be significantly associated with depression during the COVID-19 pandemic [R^2^ Adjusted = 0.320, *F* (13,290) = 10.517, *p* < 0.000]. A one unit increase in the age of early married girls indicates a 1.88 unit increase in depression among the girls, controlling the effect of all other covariates. Girls who followed Islam as their religion were 1.565 units less depressed than girls who followed *Sanatan* (Hindu) as their religion. A one unit increase in age at first marriage among the girls indicates a 1.993 unit decrease in depression among the early married girls. Again, a one unit increase in the duration of marriage is likely to decrease depression by 1.358 units among early married girls.

**TABLE 2:** Predicting depression among early married girls during the pandemic.

Variables	Simple linear regression						Multiple linear regression					
	B	β	*t*	*p*-value	95% CI		B	β	*t*	*p*-value	95% CI	
					Lower	Upper					Lower	Upper
Age	–0.246	–0.073	–1.265	0.207	–0.629	0.137	1.883	0.556	3.231	**0.001**	0.736	3.030
Religion												
*Sanatan* (Hindu)^Ref^												
Islam	–1.923	–0.176	–3.110	**0.002**	–3.140	–0.706	–1.565	–0.143	–2.833	**0.005**	–2.653	–0.478
Education	0.117	0.083	1.439	0.151	–0.043	0.276	–0.041	–0.029	–0.566	0.572	–0.183	0.101
Age at first marriage	–0.635	–0.163	–2.873	**0.004**	–1.070	–0.200	–1.993	–0.512	–3.403	**0.001**	–3.146	–0.841
Duration of marriage	0.319	0.049	0.853	0.394	–0.418	1.056	–1.358	–0.208	–2.188	**0.029**	–2.579	–0.136
Dropout												
No^Ref^												
Yes	–2.366	–0.107	–1.864	0.063	–4.865	0.132	–1.241	–0.056	–1.115	0.266	–3.431	0.949
Age of spouse	–0.211	–0.160	–2.812	**0.005**	–0.359	–0.063	–0.075	–0.056	–1.017	0.310	–0.219	0.070
Education of spouse	–0.423	–0.276	–4.993	**0.000**	–0.589	–0.256	–0.141	–0.092	–1.679	0.094	–0.306	0.024
Occupation of spouse												
Manual labor/farmer ^Ref^												
Service/business	0.177	0.018	0.319	0.750	–0.918	1.273	0.241	0.025	0.507	0.612	–0.694	1.177
Income of spouse	–0.00002	–0.311	–5.696	**0.000**	–0.0003	–0.0002	–0.00009	–0.116	–2.041	**0.042**	–0.0002	–0.000003
Marital adjustment	–0.083	–0.360	–6.702	**0.000**	–0.108	–0.059	–0.030	–0.128	–1.982	**0.048**	–0.059	0.000
Intimate partner violence	0.215	0.415	7.927	**0.000**	0.162	0.269	0.120	0.231	3.999	**0.000**	0.061	0.179
Subjective happiness	–0.339	–0.352	–6.537	**0.000**	–0.440	–0.237	–0.125	–0.130	–1.996	**0.047**	–0.248	–0.002

CI, confidence interval; Ref, reference category. Bold values are significant at 5% level of significance.

Furthermore, the income of the spouse was related to depression among early married girls, those whose spouses had more income were less likely to be depressed than those whose spouses’ incomes were low. A one unit increase in marital adjustment demonstrates a decrease in depression by 0.030 units among early married girls. Also, early married girls with a one unit increase in IPV had depression 0.120 units higher than those with no/less IPV. Moreover, a one unit increase in subjective happiness for early married girls led to a 0.125-unit decrease in depression.

### Determinants of anxiety among early married girls

Table 3 shows the SLR and MLR models with unstandardized and standardized coefficients with 95% CI. In the MLR model, the age, age at first marriage, duration of the marriage, education of spouse, IPV, and subjective happiness of the participants were found to be significantly associated with anxiety during the COVID-19 pandemic [R^2^ Adjusted = 0.342, *F* (13,290) = 11.618, *p* < 0.000]. A one unit increase in the age of early married girls indicates 1.653 units increase in anxiety among the girls, controlling the effect of all other variables. A one unit increase in age at early marriage leads to a decrease in anxiety by 1.326 units among early married girls. Therefore, the duration of marriage impacts anxiety, and a one unit increase in the duration of marriage demonstrates a 1.240 unit decrease in anxiety among the girls. Higher spousal education impacts anxiety level, and a one unit increase in spouse education reduces anxiety by 0.290 units among early married girls. Also, early married girls who faced less/no IPV faced less anxiety; their anxiety level increased by 0.120 units with a one unit increase in IPV. Moreover, anxiety level is decreased by 0.165 units with a one unit increase in subjective happiness among the girls.

**TABLE 3:** Predicting anxiety among early married girls during the pandemic.

Variables	Simple linear regression						Multiple linear regression					
	B	β	*t*	*p*-value	95% CI		B	β	*t*	*p*-value	95% CI	
					Lower	Upper					Lower	Upper
Age	0.052	0.016	0.271	0.786	–0.323	0.426	1.653	0.499	2.951	**0.003**	0.550	2.755
Religion												
*Sanatan* (Hindu)^Ref^												
Islam	–0.817	–0.077	–1.335	0.183	–2.021	0.387	–0.700	–0.066	–1.319	0.188	–1.746	0.345
Education	0.117	0.012	0.009	0.155	0.877	–0.144	–0.106	–0.700	–1.530	0.127	–0.243	0.030
Age at first marriage	–0.635	–0.203	–0.053	–0.928	0.354	–0.633	–1.326	–0.349	–2.356	**0.019**	–2.434	–0.218
Duration of marriage	0.319	0.364	0.057	0.996	0.320	–0.355	–1.240	–0.195	–2.079	**0.038**	–2.414	–0.066
Dropout												
No^Ref^												
Yes	–2.373	–0.109	–1.914	0.057	–4.814	0.067	–1.341	–0.062	–1.254	0.211	–3.446	0.764
Age of spouse	–0.117	–0.091	–1.583	0.114	–0.263	0.029	–0.017	–0.013	–0.245	0.807	–0.156	0.121
Education of spouse	–0.510	–0.341	–6.310	**0.000**	–0.669	–0.351	–0.290	–0.194	–3.594	**0.000**	–0.448	–0.131
Occupation of spouse												
Manual labor/farmer												
Service/business	0.418	0.044	0.770	0.442	–0.651	1.487	0.376	0.040	0.823	0.411	–0.523	1.275
Income of spouse	–0.0002	–0.286	–5.180	**0.000**	–0.0003	–0.0001	0.00008	–0.099	–1.762	0.079	–0.0002	0.000009
Marital adjustment	–0.087	–0.383	–7.197	**0.000**	–0.110	–0.063	–0.027	–0.118	–1.866	0.063	–0.055	0.001
Intimate partner violence	0.218	0.431	8.302	**0.000**	0.167	0.270	0.120	0.237	4.174	**0.000**	0.064	0.177
Subjective happiness	–0.381	–0.406	–7.714	**0.000**	–0.478	–0.284	–0.165	–0.176	–2.747	**0.006**	–0.284	–0.047

CI, confidence interval; Ref, reference category. Bold values are significant at 5% level of significance.

### Determinants of stress among early married girls

Table 4 indicates the SLR and MLR models with unstandardized and standardized coefficients with 95% CI. In the MLR model, the age at first marriage, education of spouse, IPV, and subjective happiness of the participants were found to be significantly associated with stress during the COVID-19 pandemic [R^2^ Adjusted = 0.289, *F* (13,290) = 9.081, *p* < 0.000]. A one unit increase in age at early marriage demonstrates a 1.370 unit decrease in stress level among early married girls. Higher spousal education impacts this stress level; a one unit increase in the spouse’s education level indicates a 0.205 unit decrease in the stress level among the girls. In addition, a one unit increase in IPV led to a 0.107 unit increase in stress level among early married girls. Moreover, early married girls with a one unit increase in subjective happiness are 0.120 units less likely to be stressed.

**TABLE 4:** Predicting stress among early married girls during the pandemic.

Variables	Simple linear regression						Multiple linear regression					
	B	β	*t*	*p*-value	95% C1		B	β	*t*	*p*-value	95% CI	
					Lower	Upper					Lower	Upper
Age	–0.425	–0.132	–2.318	**0.021**	–0.786	–0.064	1.096	0.341	1.937	0.054	–0.018	2.209
Religion												
*Sanatan* (Hindu)^Ref^												
Islam	–0.926	–0.089	–1.559	0.120	–2.059	0.243	–0.744	–0.072	–1.387	0.166	–1.800	0.312
Education	0.070	0.052	0.906	0.366	–0.082	0.221	–0.086	–0.064	–1.226	0.221	–0.224	0.052
Age at first marriage	–0.711	–0.192	–3.408	**0.001**	–1.122	–0.300	–1.370	–0.371	–2.410	**0.017**	–2.489	–0.251
Duration of marriage	0.072	0.012	0.202	0.840	–0.628	0.772	–0.835	–0.135	–1.385	0.167	–2.020	0.351
Dropout												
No ^Ref^												
Yes	–2.675	–0.127	–2.224	**0.027**	–5.041	–0.308	–1.562	–0.074	–1.446	0.149	–3.688	0.564
Age of spouse	–0.193	–0.153	–2.695	**0.007**	–0.333	–0.052	–0.102	–0.081	–1.433	0.153	–0.242	0.038
Education of spouse	–0.448	–0.308	–5.628	**0.000**	–0.604	–0.291	–0.205	–0.141	–2.513	**0.013**	–0.365	–0.044
Occupation of spouse												
Manual labor/farmer ^Ref^												
Service/business	0.156	0.017	0.296	0.768	–0.884	1.196	0.137	0.015	0.297	0.766	–0.771	1.045
Income of spouse	–0.0002	–0.265	–4.770	**0.000**	–0.0003	–0.0001	–0.00003	–0.046	–0.787	0.432	–0.0001	0.00005
Marital adjustment	–0.071	–0.322	–5.917	**0.000**	–0.094	–0.047	–0.010	–0.047	–0.720	0.472	–0.039	0.018
Intimate partner violence	0.198	0.403	7.653	**0.000**	0.147	0.249	0.107	0.218	3.684	**0.000**	0.050	0.164
Subjective happiness	–0.347	–0.381	–7.154	0.000	–0.443	–0.252	–0.194	–0.212	–3.191	**0.002**	–0.313	–0.074

CI, confidence interval; Ref, reference category. Bold values are significant at 5% level of significance.

## Discussion

This study aimed to assess the prevalence of mental health problems among early married girls in Bangladesh during the COVID-19 pandemic and to identify the possible predictors of growing mental health issues. The findings of the study suggested that the prevalence of depression, anxiety, and stress symptoms among early married girls in Bangladesh was 60.9, 74.7, and 23.7%, respectively, and these issues are more prevalent among early married girls from the *Sanatan* (Hindu) religion and among those married earlier, i.e., 14 years or under. A recent national mental health survey in 2019 indicated that the prevalence of mental health disorders among Bangladeshi children between the ages of 7 and 17 years was 12.6%; 11.5% of girls were suffering from mental health problems, of which 5.3% were experiencing anxiety and another 0.5% were enduring depression (46). A study in Bangladesh during the COVID-19 pandemic showed that the prevalence of depression, anxiety, and stress among adolescents was 67.1, 49.4, and 40.7%, respectively (28). A Chinese study showed a significant increase in mental health problems, i.e., anxiety (54.4%), sensitivity (46%), and phobia (10.1%), among adolescents during the COVID-19 pandemic (30). From the findings of the current study as well as the existing literature, it is evident that adolescents, particularly early married girls, have experienced heightened mental health problems during the COVID-19 pandemic. For unmarried adolescents, the source of this mental distress could be prolonged home confinement and isolation, uncertainty over academic career, fear of infection, and consistent exposure to negative news on electronic and social media (11, 12, 47, 48). For early married girls, on the other hand, the source of mental distress could be various stressful life events and growing household responsibilities, including the struggle to adjust to their spouse and in-laws, forced sexual relations, and the possibility of IPV (49, 50).

The findings of this study showed that age was the key predictor of the presence or absence of depression and anxiety symptoms among early married girls in Bangladesh during the COVID-19 pandemic. It is evident that the higher the age of the early married girls, the higher the presence of depression and anxiety symptoms. A study on Jordanian women indicated that young women felt remorseful about their marriage, over which they had no control, and that these marriages led them to “feelings of being completely lost” as they were barely ready, either physically or mentally, for the marriage (50). Meanwhile, Fakhari, Allahverdipour (49) concluded that early marriage adversely affected the mental health of early married girls, particularly those in their early teens, as they often struggled to adjust to the cultural norms, beliefs, and practices associated with marriage. Moreover, being unprepared for married life, the young girls did not have any knowledge about running family affairs smoothly (50); therefore, they were suddenly overburdened with household responsibilities for which they were not ready, and thus, suffered from various mental health problems including anxiety and depression. It is, therefore, necessary to address the mental health status of early married girls, whether younger or older teenagers, especially in countries where early marriage is more prevalent as a cultural practice. This will help to minimize stressful life events, including marital maladjustment and conflict, marital rape, IPV, and the possibility of loss of valuable lives.

It is evident from this study that the age at marriage and duration of marriage, rather than the age of the early married girls, that has a negative influence on their mental health; this means that higher age at marriage lowers the likelihood of depression, anxiety, and stress symptoms among these girls. Likewise, a longer duration of marriage reduces the presence of depression and anxiety symptoms among early married girls. A study conducted on early married girls in Ethiopia and Niger found a positive relationship between age at marriage and psychological wellbeing (51). John, Edmeades (51) observed that girls above the age of 15 years had better mental health, including lower depression and anxiety, as they were more ready to take on marital responsibilities, including partners’ sexual demands and childbearing and rearing duties, than those in their early teens. Another study on Pakistani women indicated that early married women showed more psychological distress than late married women, as the former found it difficult to maintain stable communication with their spouses, which negatively affected their mutual understanding and marital adjustment (25). Baysak, Yorguner (27), in contrast, found that early married women, irrespective of their age at marriage and duration of the marriage, experienced a wide range of psychiatric problems due to unexpected life events, including physical, sexual, verbal, and emotional abuse from spouses and in-laws.

Intimate partner violence significantly influenced the mental health of the early married girls; the higher the degree of IPV, the greater the presence of depression, anxiety, and stress symptoms among the participants. A study on the prevalence of domestic violence among married couples in Bangladesh during the COVID-19 pandemic suggests that women experience more domestic violence than men, and that this doubled during the pandemic (52). Like adults, girls were exposed to violence at home due to school closures, home confinement, family stress generated from job loss, financial struggles, and early marriage (53). Studies suggest that the growing prevalence of IPV during the pandemic was triggered by financial insecurity, growing debt, unmet sexual demands, frequent arguments, and prolonged home confinement with partners (15, 16, 50, 52, 54); all of these have significantly affected the mental stability of the victims of IPV (54). In order to protect early married girls against IPV, it is necessary for child protection agencies and community leaders to monitor and detect ill treatment of early married girls and to take necessary action to reduce their risk of being mistreated, whether physically or mentally.

Meanwhile, it is apparent from this study that subjective happiness among early married girls reduces their mental health burdens, including depression, anxiety, and stress symptoms. It is well documented that early married girls who are happy with their marriage do not experience traumatic mental health issues. For example, Mrayan and Obeisat (50) found that when early married girls experienced upward mobility–a change in social status and positive treatment from their spouse–they felt happy and did not report mental health issues. Another study found a direct inverse association between a supportive relationship with a partner and depression (55). The authors further noted that a supportive relationship with a partner reduces financial needs and stressful life events and enables a warm relationship with friends and other relatives (55).

Other factors that predicted the presence or absence of mental health issues among early married girls were their religion, spousal characteristics such as education, income, and marital adjustment. The findings indicate that higher spousal income reduced depression symptoms among early married girls, while higher education among spouses minimized anxiety symptoms. Likewise, Muslim early married girls reportedly experienced less depression symptoms, while better marital adjustment also reduced depression symptoms among early married girls. Generally, educated husbands with secure and stable incomes or wealth were less likely to mistreat their wives (56), and it is well evidenced that people with more supportive partners experience less mental stress, as documented by Horwitz, McLaughlin (55). However, marital adjustment, according to Shaud and Asad (25), is subject to age at marriage, as early married girls showed more deference and higher psychological stress compared to late married women. A recent study in Bangladesh suggests that housewives with stable household incomes, the lion’s share of which was contributed by their spouses, experienced fewer mental health issues during the COVID-19 pandemic (57). In this study, however, the causal relationship between spousal attributes and the marital adjustment of early married girls has not been untangled. It is also evident that compared to *Sanatan* (Hindu) early married girls, Muslim girls showed fewer depression symptoms. In the *Sanatan* (Hindu) religion, the role of women is strictly limited, i.e., submissive; dowry, e.g., “costly garments and ornaments,” is a strict custom, to be paid by the bride’s family to that of the groom (58). Demand for dowry from grooms or failure to provide dowry by the bride’s family may result in heightened depressive symptoms among early married *Sanatan* (Hindu) girls, because domestic violence and even homicide as a result of unpaid dowry is common in the Hindu community (58).

## Strengths and limitations

To interpret the results of this study, certain limitations need to be considered. Age at marriage and mental health status, i.e., depression, anxiety, and stress, were self-reported, which may be subject to recall and social desirability bias. In addition, the cross-sectional nature of the survey may limit the direct and indirect causal relationship between mental health problems and socio-demographic and marital issues. Despite these limitations, this study provides a unique contribution to the literature by assessing the prevalence and associated factors of mental health problems among early married girls during the COVID-19 pandemic. Furthermore, in this study, globally standardized and validated tools were used to measure the mental health problems, marital adjustment, IPV, and subjective happiness of early married girls. However, a more empirical study at the national level is needed to understand the mechanism.

## Conclusion and implication

This study demonstrates that early married girls have experienced heightened mental health problems and that their age, age at marriage, duration of marriage, spousal attributes including education and income, marital adjustment, IPV, and subjective happiness are the key predictors of mental health problems. From the findings, it should be clear to policymakers, community leaders, parents, and young men and women that early marriage leads to grave mental health outcomes. Therefore, they should play decisive roles in reducing the likelihood of early marriage by promoting education for girls and challenging cultural practices and social norms with harmful ramifications. These actors should raise awareness and introduce deterrents in the form of rules and regulations in order to reduce early marriage and to enable women to contribute to the national economy using their full potential. Policymakers also need to pay attention to financially poor and marginalized households, as underaged boys and girls from such families are more vulnerable to early marriage, and this may have a detrimental impact on their mental health and harm their natural cognitive development by affecting their immediate wellbeing. In addition to preventive measures against early marriage, policymakers also need to develop a support system for early married girls, with special focus on poverty-stricken household where economic insecurity together with harsh home environment affects female adolescents, in order to address the risk factors associated with early marriage. Meanwhile, it is also important to take into account the fact that after marriage the presence of financial hardship and IPV in home settings worsening female adolescent’s mental health status; therefore, necessary strategies should be placed in order to improving these girls’ social status and minimizing their mental health problems.

## Data availability statement

The raw data supporting the conclusions of this article will be made available by the authors, without undue reservation.

## Ethics statement

The studies involving human participants were reviewed and approved by the Khulna University Ethical Clearance Committee, KUECC-2022/08/24. Written informed consent to participate in this study was provided by the participants, following the verbal assent of local guardians.

## Author contributions

JN: conceptualization, investigation, data curation, and writing – original draft. T-E-AS: methodology, resources, supervision, and writing – original draft. BA and MI: data curation, formal analysis, software, and writing – original draft. MR: software and writing – original draft. MTH: conceptualization, investigation, data curation, formal analysis, methodology, resources, software, supervision, and writing – original draft. All authors writing – review and editing, contributed to the article, and approved the submitted version.

## Supplementary material

Supplementary Figure 1 Map of the study area.
