# Supplementary figures and images for: Mental health status of early married girls during the COVID-19 pandemic: A study in the southwestern region of Bangladesh
Source: Front Psychiatry. 2023 Jan 5;13:1074208. doi: 10.3389/fpsyt.2022.1074208 (PMC9849885; doi:10.3389/fpsyt.2022.1074208)

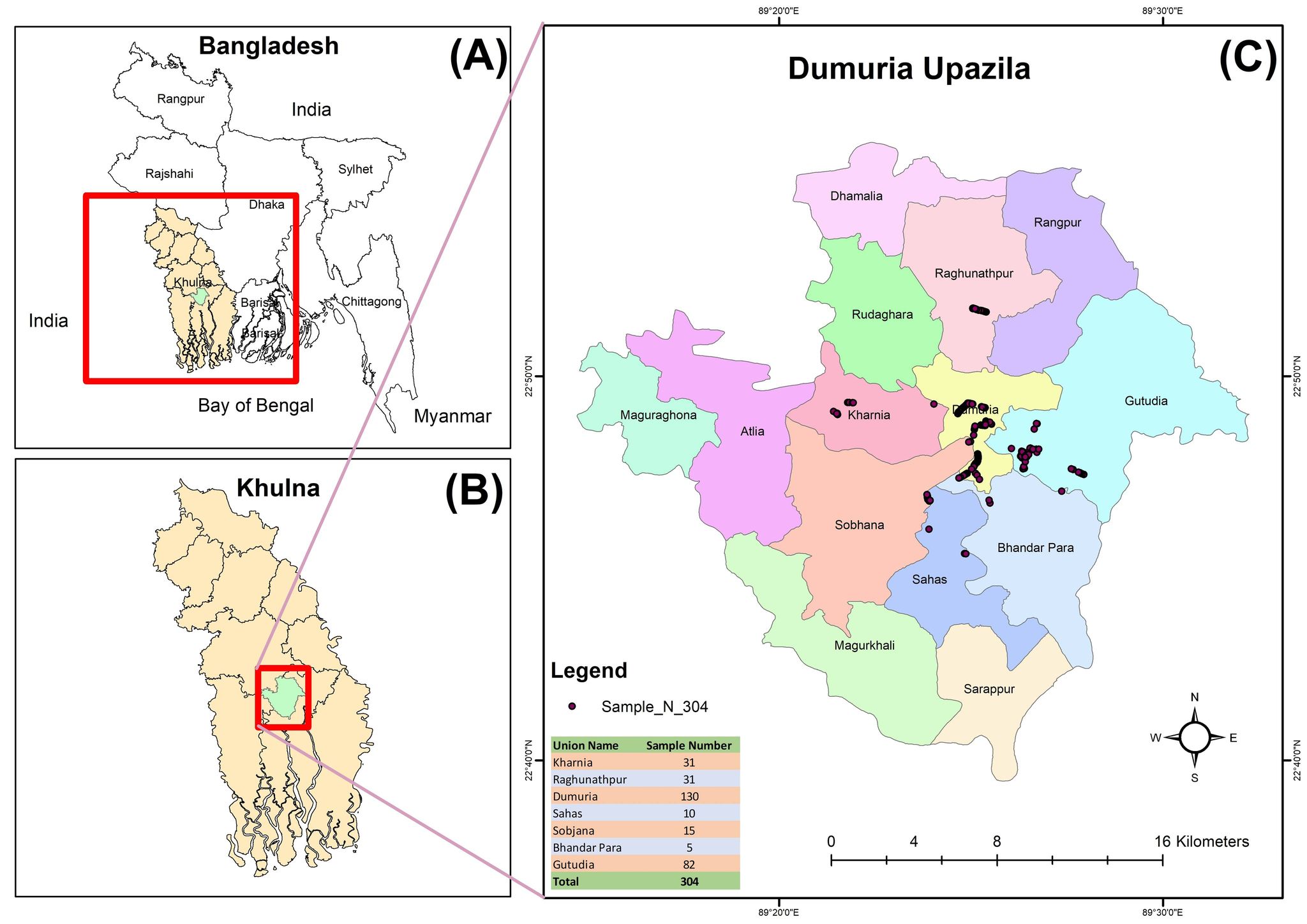

Supplement: Supplementary Figure 1 — Map of the study area. [file Image_1.JPEG]
